# Melittin Increases Cisplatin Sensitivity and Kills KM-H2 and L-428 Hodgkin Lymphoma Cells

**DOI:** 10.3390/ijms22010343

**Published:** 2020-12-31

**Authors:** Teresa Kreinest, Ines Volkmer, Martin S. Staege

**Affiliations:** Department of Pediatrics I, Martin Luther University Halle-Wittenberg, 06120 Halle, Germany; teresa.kreinest@hotmail.de (T.K.); ines.volkmer@uk-halle.de (I.V.)

**Keywords:** Hodgkin lymphoma, drug resistance, Rhodamin-123, ABC transporters, melittin

## Abstract

Hodgkin lymphoma (HL) is neoplasia with high cure rates. However, not all patients can be cured with the current treatment. Chemo-resistance of tumor cells is one factor involved in treatment failure. In addition to its pore-forming activity on lipid bilayer membranes, the toxin melittin from bee venom is an inhibitor of several cancer-related signaling pathways. Moreover, melittin analogs have been shown to inhibit the activity of ATP binding cassette (ABC) transporters which are known to play important roles in the chemo-resistance of tumor cells. Therefore, we tested the toxicity of melittin for HL cell lines KM-H2 and L-428 and whether melittin can increase the chemo-sensitivity of cisplatin-resistant HL cells. We found high toxicity of melittin for KM-H2 and L-428 cells. In co-cultures with normal blood cells, melittin preferentially killed KM-H2 and L-428 cells. In addition, we observed increased cisplatin sensitivity of chemo-resistant L-428 cells after treatment with melittin. ABC transporter activity was not reduced after treatment with melittin. Our data suggest that melittin or melittin analogs might be promising agents for the future development of treatment strategies for HL patients with resistant disease.

## 1. Introduction

Hodgkin lymphoma (HL) is hematopoietic neoplasia which can be cured in most cases. However, a small percentage of tumors do not respond adequately to the currently available therapy and the prognosis for patients with resistant disease or relapse is unsatisfying. The pathogenesis of HL has not been clarified completely but aberrant activation of several signaling pathways has been described (reviewed in [1,2]). Melittin (MEL) is a pore-forming peptide from bee venom. Anti-cancer activities of MEL have been proposed 50 years ago [3]. At the same time, MEL has been described as a putative tumor promoter with similar effects as phorbol esters [3,4,5,6]. The tumor promoter-like activities have been interpreted as a consequence of altered membrane permeability for sodium and activation of phospholipase A2 (PLA2) by MEL [6,7]. PLA2 activation and subsequent production of arachidonic acid have been proposed as a mechanism for the toxicity of MEL for tumor cells [8]. Interestingly, MEL shows growth inhibitory activity and high toxicity for hematopoietic tumor cells. [9,10,11]. Several other mechanisms have been proposed for the toxicity of MEL for tumor cells, including inhibition of calmodulin by MEL [10,11,12,13]. In addition, pore formation by MEL in Burkitt lymphoma cells has similar apoptosis initiating effects as the complement membrane attack complex [14]. MEL can also increase the toxicity of cytostatic drugs for normal hematopoietic cells [15]. However, the sensitivity of malignant cells for MEL was shown to be significantly higher than the sensitivity of normal hematopoietic cells [16]. Similar differences in the sensitivity were observed in solid tumor cells and their normal counterparts [17]. In order to reduce toxicity for normal cells and increase specificity, immunoconjugates, and pro-drugs that release MEL at the site of tumor cells have been developed [18,19]. In such conjugates, MEL can be replaced by synthetic MEL variants with increased lytic activity [20,21,22]. In addition, less toxic MEL analogs have been synthesized which retain anti-cancer activities [23,24,25,26,27,28,29,30,31,32]. MEL showed high toxicity for cells with activated *RAS* oncogene and MEL resistance was paralleled by down-regulation of RAS [33]. This effect was a consequence of the influx of Ca^2+^ into the tumor cells and strong PLA2 activation [34]. In other models, MEL-mediated Ca^2+^ influx was shown to be independent of PLA2 [35]. Interestingly, MEL mediated Ca^2+^ influx was shown to induce cross-protection against other pore-forming molecules including complement and perforin [36]. As a peptide toxin, MEL can be introduced as a transgene in cells. Transgenic expression of MEL in bladder carcinoma cells or hepatocellular carcinoma demonstrated anti-tumor efficacy in vitro and in xenograft models [37,38,39]. In leukemia cells, MEL can increase tumor necrosis factor (TNF) toxicity by activation of PLA2 [40]. In addition to PLA2, PLD is activated by MEL in leukemia cells [41] and it seems that membrane disruption by MEL results in activation of multiple lipases [42]. Several cancer-related signaling pathways are targeted by MEL and MEL analogs. These include inhibition of the AKT pathway [43], inhibition of the RAC1 pathway [44], and inhibition of the NFKB pathway [45,46,47,48,49]. The importance of such pathways in HL suggest that melittin might also be effective in these cancer cells. Therefore, we analyzed the effects of MEL on the two HL cell lines KM-H2 and L-428.

## 2. Results

### 2.1. Melittin is Toxic for KM-H2 and L-428 HL Cells

**Figure 1:**
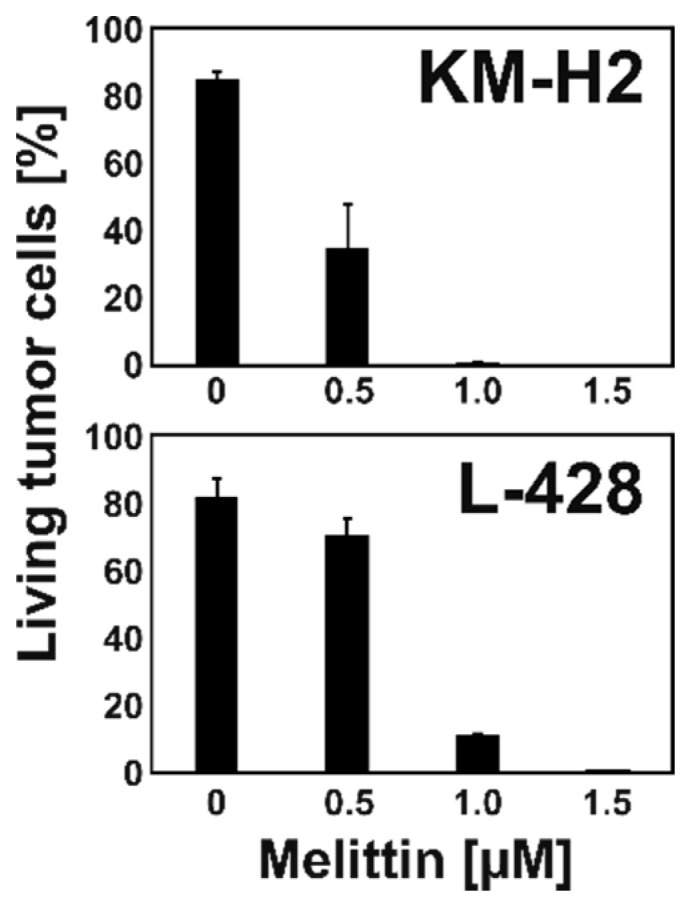
Toxicity of MEL for HL cell lines KM-H2 and L-428. HL cells were incubated together with PBMC in the presence or absence of different concentrations of MEL. Cell viability was assessed by flow cytometry. Presented are viable tumor cells as percentages of all living cells. Means and standard deviations from two independent experiments.

**Figure 2:**
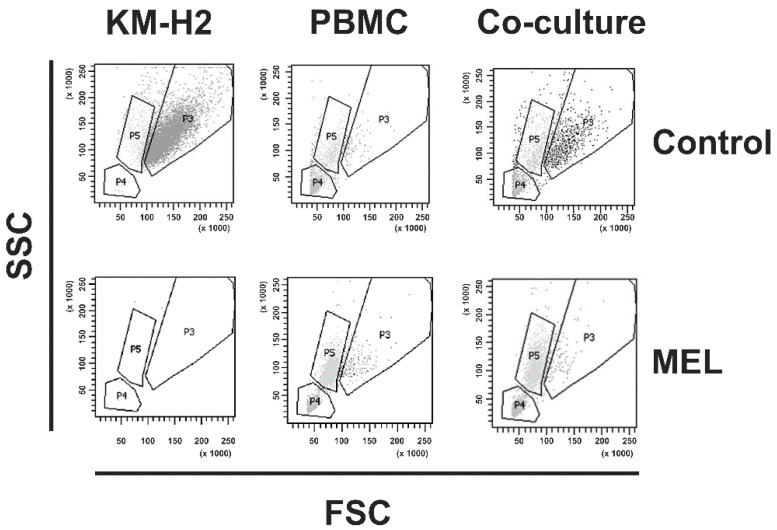
MEL increases monocyte numbers. KM-H2 cells, PBMC, and co-cultures of KM-H2 cells and PBMC were incubated with 1.5 µM MEL or medium without MEL. Thereafter, cells were stained with propidium iodide and analyzed by flow cytometry. Presented are representative dot blots of PI negative living cells. Note that nearly no living cells were visible in KM-H2 cells after treatment with MEL. SSC: side scatter; FSC: forward scatter.

**Figure 3:**
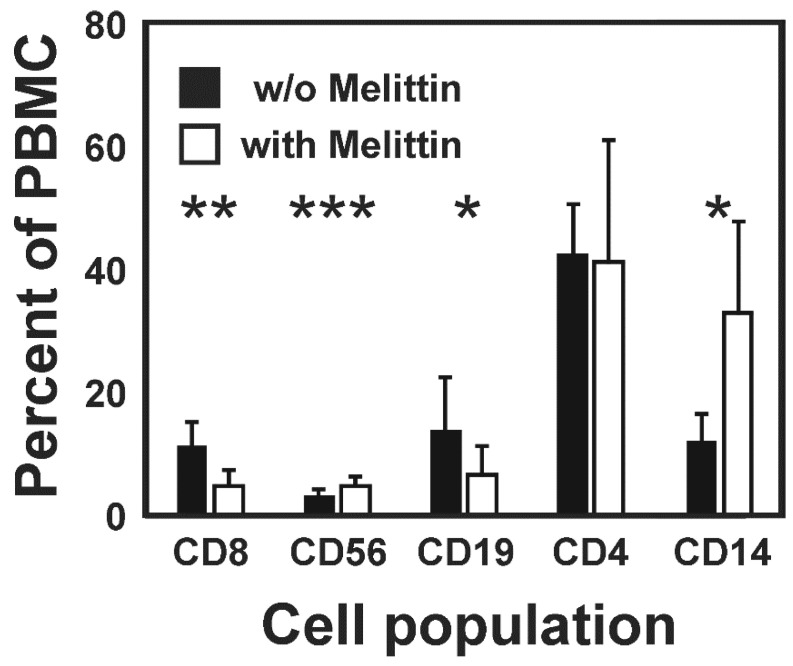
Changes in blood cell populations after treatment with MEL. PBMC were treated with 1.5 µM MEL or left untreated and analyzed by flow cytometry using antibodies directed against CD3, CD4, CD8, CD14, and CD56. Presented are means and standard deviations for the different cell populations from four independent experiments. Asterisks indicate statistical significance (*: *p* < 0.05; **: *p* < 0.01; ***: *p* < 0.001; Student’s *t*-test).

We observed high toxicity of MEL for HL cell lines KM-H2 and L-428. IC50 values were found to be 0.93 +/− 0.42 µM for L-428 cells and 0.75 +/− 0.073 µM for KM-H2 cells (see Supplementary Figure S1). The toxicity of MEL for these HL cells was higher than the toxicity for normal blood cells. We co-cultured KM-H2 or L-428 cells with normal peripheral blood mononuclear cells (PBMC) in the presence of different concentrations of MEL. HL cells and PBMC were discriminated using their different cell size. As shown in Figure 1, the percentage of living tumor cells decreased with increasing MEL concentrations. Interestingly, we observed a shift in the composition of the surviving normal PBMCs. As shown in Figure 2 we observed a strong increase in the monocyte population. This increase was also visible in cultures without HL cells. After staining with specific antibodies, we observed a strong increase in the percentage of CD14-positive monocytes and a less pronounced but significant increase in the percentage of CD56-positive NK cells (Figure 3). Percentages of CD19-positive B cells and CD8-positive cytotoxic T cells decreased whereas CD4-positive T helper cells remained unchanged.

### 2.2. Melittin Increases Cisplatin Sensitivity of Chemo-Resistant L-428 Cells

**Figure 4:**
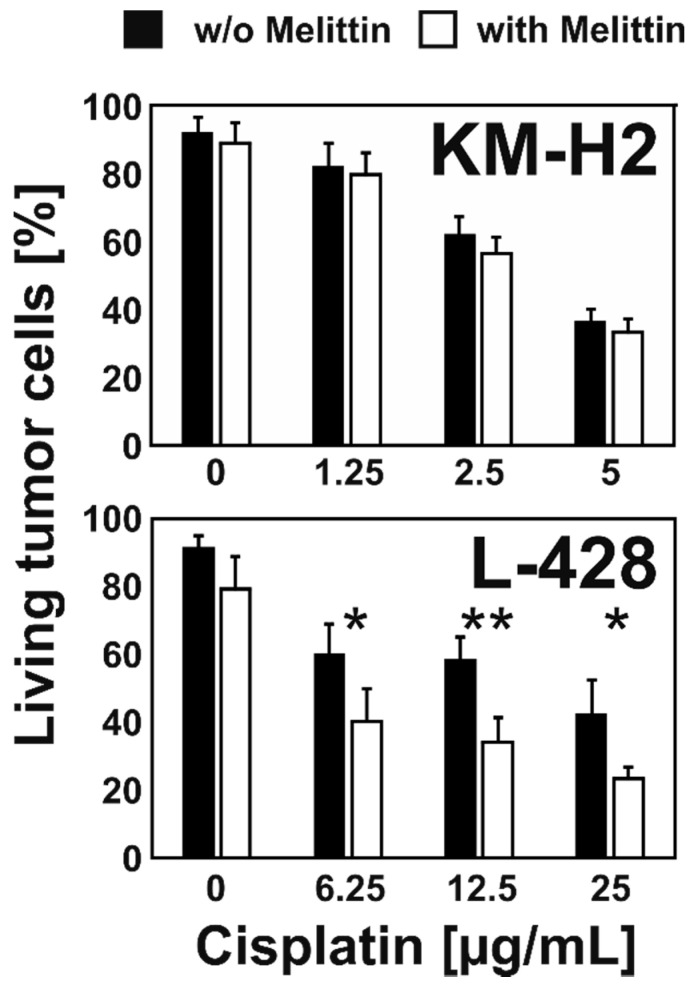
MEL increases cisplatin sensitivity of resistant L-428 cells. L-428 cells and KM-H2 cells were treated with different concentrations of cisplatin after a 72-h pre-treatment period with 0.5 µM MEL or without MEL. Viability was assessed by flow cytometry. Presented are means and standard deviations from four (L-428 cells) or three (KM-H2) independent experiments. Asterisks indicate statistical significance (*: *p* < 0.05; **: *p* < 0.01; Student’s *t*-test).

We asked whether the combination of MEL with cytotoxic drugs could enhance the drug sensitivity of resistant HL cells. Therefore, we pre-incubated L-428 cells with a low concentration of MEL and tested sensitivity for cisplatin. As shown in Figure 4, MEL pre-treated L-428 cells showed significantly enhanced sensitivity for cisplatin. In KM-H2 cells, which have a higher basal sensitivity for cisplatin (see Supplementary Figure S2), only marginal effects were observed (Figure 4).

### 2.3. Melittin Has no Influence on ABC Transporter Activity of KM-H2 and L-428 Cells

**Figure 5:**
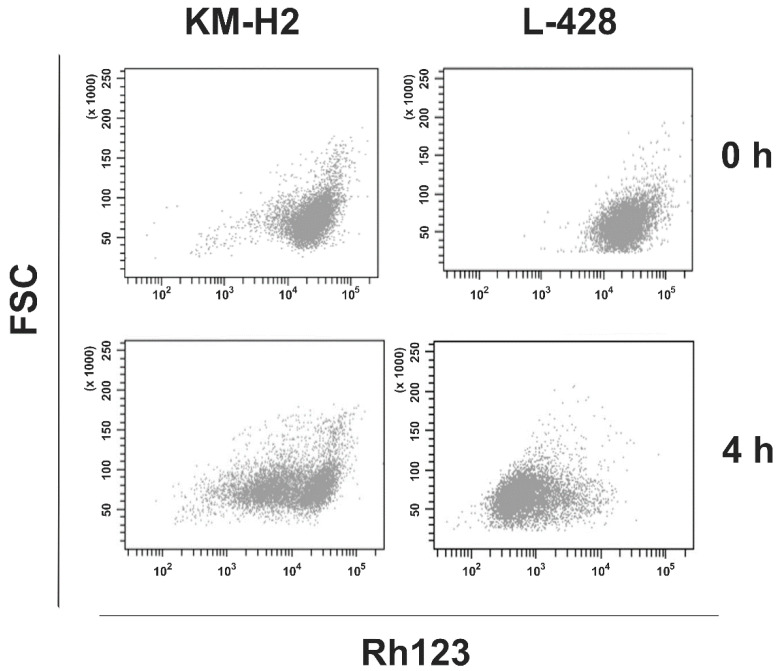
Rh123 efflux from HL cell lines KM-H2 and L-428. Cells were stained with Rh123 and incubated for 4 h at 37 °C. Rh123 staining was assessed by flow cytometry.

**Figure 6:**
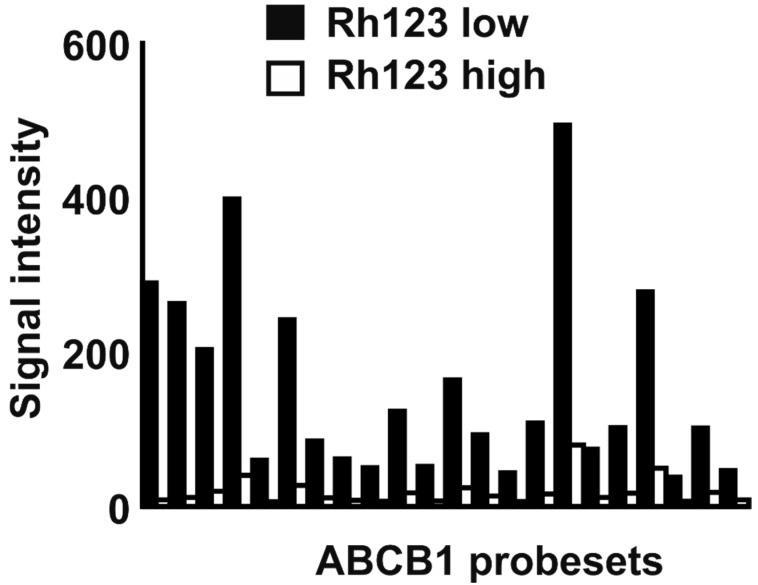
Higher expression of *ABCB1* in KM-H2 cells with higher Rh123 efflux capacity. KMH2 cells with high Rh123 fluorescence after 4 h efflux time (Rh123 high) and with low Rh123 fluorescence were isolated by flow cytometry and global gene expression was analyzed by DNA microarray analyses. Presented are signal intensities from ABCB1 specific probe sets in the two cell populations.

**Figure 7:**
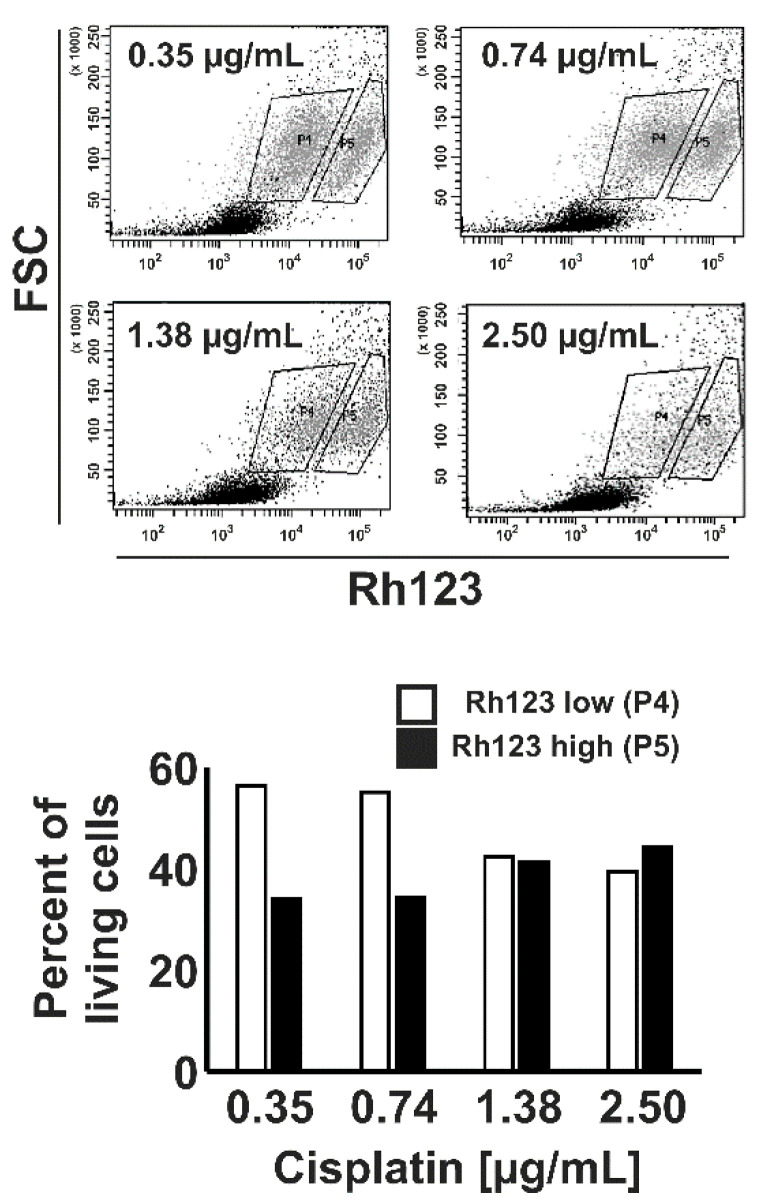
KM-H2 cells with high Rh123 efflux capacity are not resistant to cisplatin. KM-H2 cells were incubated with increasing concentrations of cisplatin for 24 h. Thereafter, cells were stained with Rh123 and dye efflux was assessed after 4 h by flow cytometry. The percentages of living cells with high and low Rh123 staining were determined.

**Figure 8:**
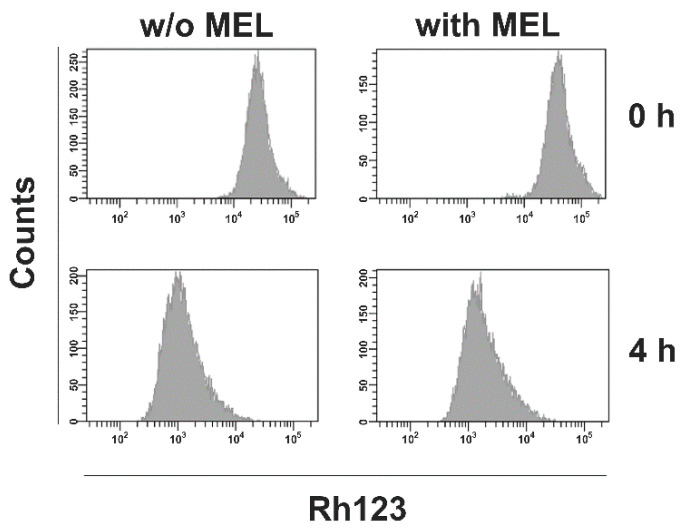
MEL has no effect on ABC transporter activity in L-428 cells. L428 cells were incubated for 48 h with 0.5 µM MEL or without MEL (w/o MEL). Thereafter, cells were stained with Rh123 and dye efflux was assessed after 4 h by flow cytometry.

Recently it was shown that a MEL analog can inhibit expression of the multidrug resistance ABC transporter *ABCB1* [48]. ABCB1 activity can be measured by quantification of efflux of the dye Rhodamin-123 (Rh123). We observed higher ABCB1 transporter activity in the chemo-resistant HL cell line L-428. Interestingly, KM-H2 cells showed two populations of cells with different Rh123 efflux activity (Figure 5). We sorted these cells by flow cytometry. DNA microarray analysis indicated that KM-H2 cells with high Rh123 efflux capacity had higher expression of *ABCB1* (Figure 6). We asked whether ABCB1 activity influences drug sensitivity in HL cell lines. After incubation of KM-H2 cells with cisplatin, we observed no selective depletion of Rh123 high cells (Figure 7). In addition, we observed no increased Rh123 efflux in L-428 cells after treatment with MEL (Figure 8). These observations suggest that ABCB1 transporter activity plays no major role in the drug sensitivity of these cells. Accordingly, ABC transporters were not among the top genes that we identified in our previous gene expression profiling studies as resistance-associated genes [50].

## 3. Discussion

In the present study, we observed the killing of L-428 and KM-H2 HL cells by MEL and increased sensitivity of MEL-pre-treated L-428 cells for cisplatin. MEL has been shown to have anti-neoplastic but also anti-bacterial and anti-viral activities [51,52]. These effects can partially be explained by the membrane disrupting activity of MEL. However, MEL targets multiple cellular signaling pathways that are altered in cancer cells or during microbial infections. One of these pathways is the NFKB pathway [46,47,48,49]. This pathway is involved in inflammatory and neoplastic processes [53,54]. Anti-inflammatory effects of MEL have been described [55,56,57,58]. On the other hand, as a peptide antigen melittin might induce unwanted antibody responses. Shorter melittin analogs have been shown to have low immunogenicity [59].

HL is neoplasia with complex interaction between the tumor cells and the non-malignant bystander cell compartment. HL cells can be attacked by the immune system but the interaction between HL cells and the immune system is obviously insufficient. Low expression of major histocompatibility class II antigens on HL cells has been observed in more than 40% of cases and was found to be an adverse prognostic factor [60]. HL cells often express immune-regulatory molecules like the programmed cell death protein 1 ligand [61] and novel therapeutic strategies can use these molecules as targets [62]. Immune cells are also involved in apoptosis inhibition and growth of HL cells in vivo. Computer simulations indicate that depending on the balance between these two activities, tumor growth or remission can occur [63]. In this light, our observation of altered cell populations after blood cell exposure to MEL might be important. The importance of the monocyte/macrophage compartment on HL biology has been described [64]. Monocytes can be used for the preparation of dendritic cell-based vaccines and it might be interesting to investigate the effect of MEL on the function and phenotype of monocytes in this context.

L-428 cells are resistant to conventional cytotoxic drugs. Interestingly, these cells also show a relatively low sensitivity for MEL compared to the more sensitive KM-H2 cell line (see Figure 1). In our previous investigations, we observed resistance of HL cell lines against multiple drugs, suggesting that resistance is not specific for single drugs but a consequence of general resistance mechanisms and anti-apoptotic mechanisms [50]. Intracellular accumulation of cytotoxic drugs is a necessary step for drug action. Interestingly, L-428 cells have high Rh123 efflux activity suggesting that these cells have impaired intracellular accumulation of cytotoxic drugs. Cisplatin is not a known substrate for ABCB1, but ABCB1 overexpression is often associated with cisplatin resistance and ABCB1 has anti-apoptotic activities that are independent of the pump function [65,66]. However, we saw no increased cisplatin sensitivity for HL cells with low ABCB1 activity and in our previous gene expression profiling studies, ABC transporters were not associated with chemo-resistance of HL cells [50]. Moreover, pre-treatment with MEL has no effect on ABC transporter activity in KM-H2 and L-428 cells and cisplatin showed no increased efficiency against cells with low ABC transporter activity. All these observations suggest that ABC transporters are not key players in the chemo-resistance of HL cells. This is in agreement with earlier studies that found ABC transporter expression on bystander cells but not neoplastic cells in Hodgkin lymphoma biopsies [67]. Similarly, *ABCB1* gene polymorphisms have been associated with the occurrence of HL but not with the response to therapy [68].

It seems likely that the targeting of other pathways by MEL is responsible for the increased toxicity. The NFKB pathway is one candidate for such activity because this pathway is critically involved in HL pathogenesis and is a putative target of MEL. However, preliminary results suggest that neither NFKB nor NFKB target genes were up-regulated in L-428 cells after treatment with MEL (see Supplementary Figure S3). The NFKB pathway might not be targeted by MEL in all cell types as similar results were described for synoviocytes, fibroblasts, and blood cells [69]. Future studies should elucidate the specific activities of MEL on signaling pathways in HL. Moreover, the combination of MEL and cisplatin might have synergistic effects that are different from the effects of single drugs. For instance, it was shown that the metabolome of ovarian cancer cells after combination treatment differs from the metabolome of cells treated with MEL or cisplatin alone [70]. Such effects could be relevant for future therapeutic developments. Reducing therapy toxicity is a goal of many studies in the field of oncology. Interestingly, low doses of cytotoxic drugs can have anti-cancer activities that are independent of the cytotoxic activity [71]. However, low drug concentrations may also promote the development of drug resistance. For example, it has been shown that in a xenograft model, low concentrations of cisplatin lead to the emergence of cisplatin-resistant cancer cells [72]. If combination therapies target other pathways than the respective drugs alone, the acquisition of resistance to the individual drugs could be overcome or at least delayed.

Some limitations of our study should be claimed. Most importantly, we have not systematically determined the optimal treatment schedule for the combination of MEL and cisplatin. Further experiments are needed in order to establish the best combination protocol.

In conclusion, our data suggest that MEL or MEL analogs might be interesting for future interventions for the treatment of HL. The elucidation of the mechanism behind MEL-induced sensitization of L-428 cells for cisplatin can lead to new insight into drug resistance of HL cells. To this end, changes in the global transcriptome or proteome of MEL treated cells should be investigated. The (sequential or simultaneous) combination of MEL with conventional drugs might also be extended to other drugs currently used for the treatment of HL patients.

## 4. Materials and Methods

### 4.1. Cells and Cell Culture

HL cell lines L-428 [73] and KM-H2 [74] were obtained from the German Collection of Microorganisms and Cell Cultures (Braunschweig, Germany) and were cultured in RPMI medium supplemented with 10% FCS and 1% penicillin/streptomycin (10,000 U/mL/10,000 µg/mL; Gibco, Thermo Fisher Scientific, Waltham, MA, USA). PBMC were isolated as described [75].

### 4.2. Rh123 Staining and Flow Cytometry

L-428 cells and KM-H2 cells were harvested and resuspended in 5 mL RPMI Medium. The cells were stained with 0.2 µg/mL Rh123 (Sigma, Heidelberg, Germany) and incubated for 30 min. Cells were washed and incubated for 4 h at 37 °C. After the incubation time, the stained cells were prepared for flow cytometry. The stained cells were harvested and centrifuged for 10 min at 200× *g*. The pellet was resuspended in 1 mL phosphate-buffered saline (PBS) and analyzed on an LSR II cytometer (BD Biosciences, Franklin Lakes, NJ, USA). For antibody staining, the cells were harvested, and the cell pellet was resuspended in 50 µL PBS. Then 10 µL of the antibody solution was added. After incubation for 30 min at 4 °C, the cells were centrifuged for 10 min at 200× *g* and the pellet was resuspended in 1 mL PBS and analyzed on an LSR II cytometer. Fluorochrome labeled antibody combinations directed at CD3/4, CD5/19, CD8/56/3, and CD45/14 were purchased from BD Biosciences (Heidelberg, Germany). For each sample between 5 × 10^5^ and 10^6^ cells were used and data for 10^4^ cells were collected.

### 4.3. Melittin and Cisplatin Treatment

For the pre-treatment with melittin, cells were treated with 0.5 µM of melittin (Sigma, Heidelberg, Germany) for 72 h at 37 °C. This concentration was used because in preliminary experiments we observed that the majority of the cells survive this concentration (see Supplementary Figure S1). Thereafter, cells were transferred into a 24-well plate at 200,000 cells per well. Cells were treated with different concentrations of cisplatin (Sigma, Heidelberg, Germany). The concentrations which were used for the treatment of the KM-H2 cell line are 5 µg/mL, 2.5 µg/mL, 1.25 µg/mL, and 0 µg/mL as a control. The concentrations which were used for the treatment of the more resistant L-428 cell line are 25 µg/mL, 12.5 µg/mL, 6.25 µg/mL, and 0 µg/mL as a control. All cultures received the same volume of drug solution or dimethylformamide (DMF) as solvent. The cells were incubated with cisplatin for 24 h at 37 °C. After incubation, the cells were prepared for flow cytometry analysis. Therefore, the cells were resuspended and transferred into flow cytometry tubes. After centrifugation at 200× *g* for 10 min, the supernatant was removed, and the cell pellet was resuspended in 1 mL PBS. Immediately before analysis, the cells were stained with 10 µL propidium iodide (Sigma, Heidelberg, Germany) for the detection of dead cells.

### 4.4. Co-Culture Experiments

For co-culture experiments, cells of the cell lines KM-H2 and L-428 were harvested and seeded at 250,000 tumor cells per well in 24-well plates. 1.25 Million PBMC per well were added. Tumor cells alone and PBMC alone served as controls. All cells were treated with different concentrations of melittin. The concentrations which were used for these treatments are 1.5 µM, 1 µM, 0.5 µM, and 0 µM as a control. After 24 h incubation at 37 °C, the cells were prepared for flow cytometry analysis. Therefore, the cells were resuspended and after centrifugation at 200× *g* for 10 min, the supernatant was removed, and the cell pellets were resuspended in 1 mL PBS. Immediately before analysis, the cells were stained with 10 µL propidium iodide.

### 4.5. DNA Microarray Analysis

DNA microarray analysis was performed using Clariom D Human arrays (Thermo Fisher Scientific, Waltham, MA, USA). Data analysis was performed with the Transcription Analysis Console (Thermo Fisher Scientific, Waltham, MA, USA).

## Data Availability

Raw microarray data will be available from the Gene Expression Omnibus data base (https://www.ncbi.nlm.nih.gov/gds).

## Supplementary Materials

The following are available online at https://www.mdpi.com/1422-0067/22/1/343/s1, Figure S1: Melittin toxicity for L-428 and KM-H2 cells. Figure S2: Cisplatin toxicity for L-428 and KM-H2 cells. Figure S3: Expression of NFKB and target genes in MEL-treated L-428 cells.

## Abbreviations

ABC	ATP binding cassette
HL	Hodgkin Lymphoma
MEL	Melittin
NFKB	Nuclear factor kappa-light-chain-enhancer of activated B-cells
Rh123	Rhodamin-123
