# Supplementary material for: Melittin Increases Cisplatin Sensitivity and Kills KM-H2 and L-428 Hodgkin Lymphoma Cells
Source: Int J Mol Sci. 2020 Dec 31;22(1):343. doi: 10.3390/ijms22010343 (PMC7795649; doi:10.3390/ijms22010343)
Supplement: Supplementary file 1 [file ijms-22-00343-s001.pdf]

Supplementary Material for:

Melittin Increases Cisplatin Sensitivity and Kills KM-H2 and L-428 Hodgkin Lymphoma Cells

Teresa Kreinest, Ines Volkmer and Martin S. Staege

Content:

Figure S1 page 2

Figure S2 page 3

Figure S3 page 4

Supplementary Figure S1

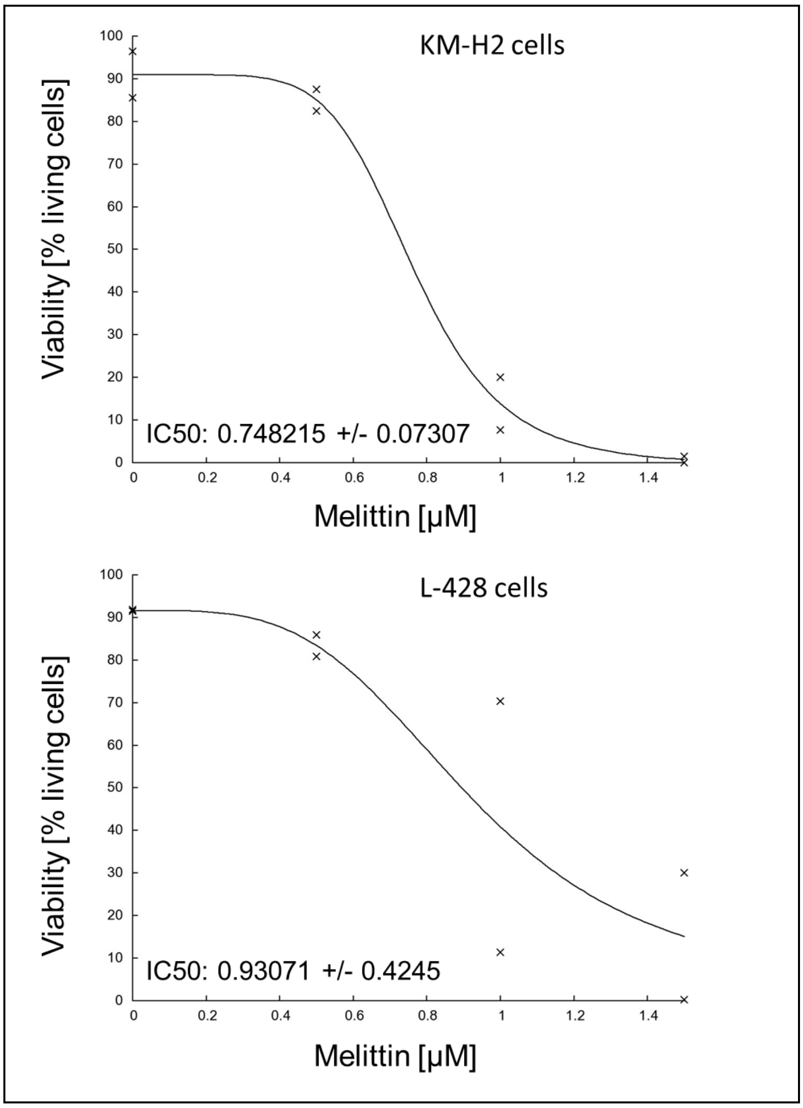

Figure S1. Melittin toxicity for L-428 and KM-H2 cells. L-428 cells and KM-H2 cells were treated for 72 hrs with varying concentration of melittin. Thereafter, cells were stained with propidium iodide and viability was assessed by flow cytometry. Data are from two independent experiments. IC50 values and regression curves were calculated with the IC50 tool kit (http://www.ic50.tk/index.html).

Supplementary Figure S2

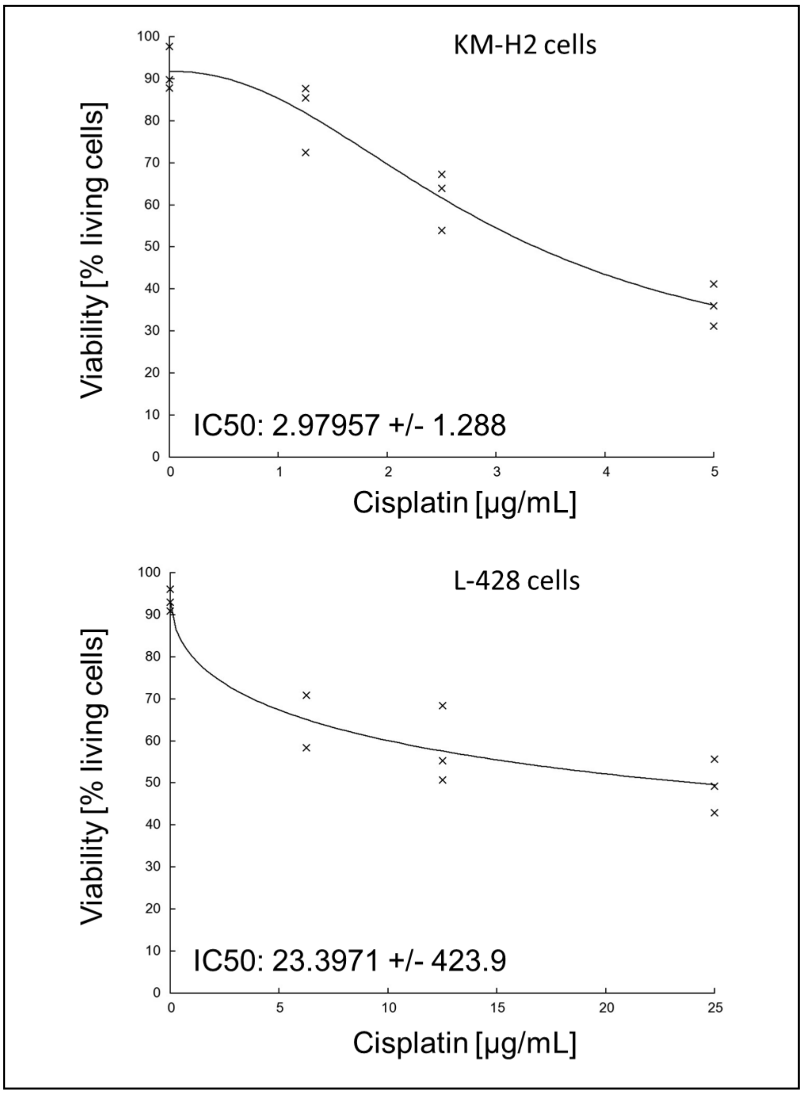

Figure 2. Cisplatin toxicity for L-428 and KM-H2 cells. L-428 cells and KM-H2 cells were treated for 24 hrs with varying concentration of cisplatin or the same volume of DMF as control. Thereafter, cells were stained with propidium iodide and viability was assessed by flow cytometry. Data are from 3 independent experiments. IC50 values and regression curves were calculated with the IC50 tool kit (http://www.ic50.tk/index.html).

Supplementary Figure S3

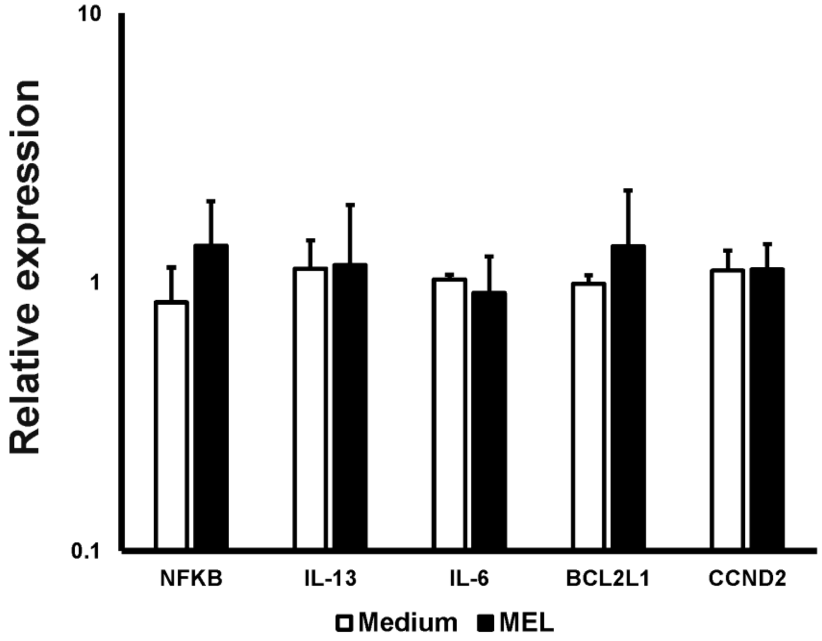

Figure 3. Expression of NFKB and target genes in MEL-treated L-428 cells. L-428 cells were treated for 72 hrs with 0.5 µM MEL. Thereafter, RNA was isolated, reverse transcribed into cDNA and subjected to quantitative PCR. The following primer combinations were used: B cell leukemia/lymphoma 2 like 1 (BCL2L1): 5'-CAT GGC AGC AGT AAA GCA AG-3' and 5'- GCA TTG TTC CCA TAG AGT TCC-3'; cyclin D2 (CCND2): 5'-TGG GGA AGT TGA AGT GGA AC-3' and 5'-ATC CAC GTC TGT GTT GGT GA-3'; interleukin 6 (IL-6): 5'-CTC ACC TCT TCA GAA CGA ATT G-3' and 5'-CCA TCT TTG GAA GGT TCA GGT TG-3'; interleukin 13 (IL-13): 5'-CCT CAT GGC GCT TTT GTT GAC-3' and 5'-TCT GGT TCT GGG TGA TGT TGA-3'; nuclear factor kappa B subunit 1 (NFKB) 5'-CAC CTA GCT GCC AAA GAA GG-3' and 5'-TCA GCC AGC TGT TTC ATG TC-3'. Hypoxanthine phosphoribosyltransferase 1 (primer sequences: 5'-ACC AGT CAA CAG GGG ACA TAA-3' and 5'-CTT CGT GGG GTC CTT TTC ACC-3') was used as housekeeping control and the median off all samples was set as 1. Relative expression was calculated according to standard $2^{-\Delta\Delta Ct}$ method. Presented are means and standard deviations from triplicate determinations.
